# Publication Productivity in Central Asia and Countries of the Former Soviet Union

**DOI:** 10.5195/cajgh.2016.261

**Published:** 2016-12-13

**Authors:** Shalkar Adambekov, Sholpan Askarova, Sharon C. Welburn, Sharon L. Goughnour, Ayumi Konishi, Ronald LaPorte, Faina Linkov

**Affiliations:** 1 Graduate School of Public Health, University of Pittsburgh; 2 National Laboratory Astana, Nazarbayev University; 3 East Asia Department, Asia Development Bank; 4 Department of Obstetrics, Gynecology & Reproductive Sciences, Magee-Womens Research Institute

**Keywords:** Publication productivity, scientific publications, Central Asia, Kazakhstan

## Abstract

**Introduction:**

Despite the significant number of research institutions and rich scientific heritage, published research from Central Asia (Kazakhstan, Uzbekistan, Kyrgyzstan, Tajikistan, and Turkmenistan) is traditionally underrepresented in international scientific literature. The goal of this paper was to analyze publication patterns in Central Asian countries, and to explore the factors that contributed to the publication productivity in Kazakhstan.

**Methods:**

Publication productivity was evaluated using data generated by the SCImago Journal & Country Rank over the period of 1996–2014 for all of the 15 former Soviet Union Republics for all subject categories. Country specific data, including total population, gross domestic product (GDP) per capita, research and development (R&D) expenditure (% of GDP), number of reserchers (per million people), was abstracted from World Bank data. ANOVA and ANCOVA analyses compared the mean number of publications among Central Asian countries. Separate analyses was done for publication patterns in the health sciences. Multiple comparisons were performed using Tukey method

**Results:**

The analysis of publication productivity showed significant discrepancies in the number of published documents among the Central Asian countries. Kazakhstan demonstrated a significant increase in the number of published documents in the period of 1996–2014, mainly in the areas of natural and multidisciplinary sciences. Our analyses also showed that the number of publications are siginicantly associated with GDP and population size.

**Conclusions:**

We identified large gaps in publication productivity among the Central Asian countries. The association between publication rate with GDP and population size indicates there is a need to adjust for these factors when planning research policy.

Scientific research conducted in Central Asia is underrepresented in the international scientific literature. Central Asia is the region consisting of the former Soviet Union Republics of Kazakhstan, Kyrgyzstan, Tajikistan, Turkmenistan, and Uzbekistan. All of these countries gained independence after the dissolution of the Soviet Union in 1991. Despite their ethnic and economic diversity, all of the “stans” share common cultural and historical roots.^1^ The economic crisis that followed the dissolution of the Soviet Union led to the degradation of the established Soviet Era scientific research infrastructure, leaving researchers struggling to compete in a more opened and integrated scientific world.^2^–^4^

As with most other former Soviet Republics, much of the research conducted in Central Asia is government controlled, and research articles are mainly published in local periodicals in the Russian language. These periodicals are generally unknown outside the former Soviet Union and are not cross referenced in research databases commonly used by global scientists, such as Scopus, Web of Science, Medline, Embase, etc. Therefore, scientific research from these countries is underrepresented in the international literature, despite notable achievements in the fundamental areas of science, such as physics, chemistry, mathematics, etc.^5^ Therefore, the aim of this manuscript was to examine publication productivity of the Central Asian region by analyzing the number of articles published by each country for the past two decades. Given that the crude number of publications and citation indexes are almost uniformly utilized as the major criteria for publication productivity evaluation both within and across countries,^6^ our study fills an important gap in our understanding of publication productivity in Central Asia.

Since most of the journals in Central Asian countries are available in print only and not indexed in any major databases,^5^,^7^ only articles that are accessible to global scientists and indexed in Scopus were analyzed. Consequently, the data for this study was obtained by analyzing information generated by the SCImago Journal & Country Rank .^8^ This online software provides a free and easy to access tool for comparing the total number of existing Scopus referenced journals, publications, and research areas of various countries in different scientific disciplines for the period from 1996 to 2014.

Detailed analyses of publication productivity trends in Kazakhstan and Central Asia have not been performed to date, a significant gap that our research is aiming to close. The goal of this study was to perform a retrospective analysis of publication patterns in Central Asian countries between 1996 and 2014 and provide a foundation for improving publication productivity in the region.

## Methods

Data on the number of publications and the number of journals existing in the region was collected from SCImago Journal & Country Rank.^8^ We analyzed country rankings and the number of published documents for the period of 1996–2014 in the subject areas of “All” and “Medicine.” Data from “Country search” was collected for 15 former Soviet Union Republics (Armenia, Azerbaijan, Belarus, Estonia, Georgia, Kazakhstan, Kyrgyzstan, Latvia, Lithuania, Moldova, Russia, Tajikistan, Turkmenistan, Ukraine, and Uzbekistan). Analysis of subject area “Medicine” was performed on countries of the Central Asian region, including Kazakhstan, Uzbekistan, Kyrgyzstan, Tajikistan, and Turkmenistan in the following categories: “Medicine,” “Public health, environmental and occupational health,” and “Epidemiology.” The number of journals published in each of the 15 post-Soviet Republics was identified through “Journal search” option in the SCImago Journal & Country Rank database.

### Data analysis

Descriptive statistics were used to evaluate the data. Latest available complete data on gross domestic product (GDP) per capita, based on purchasing power parity (PPP), research and development (R&D) expenditure (% of GDP), total population in the country (population size), and number of researchers in R&D were collected from the World Bank^9^ for each country included in the study. Number of publications, GDP, and population size were log transformed. One-way ANOVA analyses compared mean numbers of publications of Central Asian countries, and ANCOVA was used to compare the mean numbers of publications of Central Asian countries after controlling for GDP and population size. Multiple comparisons were performed using Tukey method. All analyses were conducted in SAS version 9.4 (SAS Institute Inc., Cary, NC).

## Results

Of the former 15 Soviet Union Republics, Russia and Ukraine produced the highest number of Scopus referenced documents in the subject area “All” for the period of 1996–2014, publishing 701,029 and 133,650 manuscripts, respectively. Of the remaining 13 countries, Lithuania produced the most publications with 32,137 articles (Table 1). Central Asian countries published the lower number of publications compared to other former Soviet Union Republics, with Kazakhstan leading the Central Asian region with 9,652 published articles. Turkmenistan has the lowest number of publications referenced by Scopus in Central Asian region with 284 documents produced during the period of 1996–2014.

**Figure 1.**
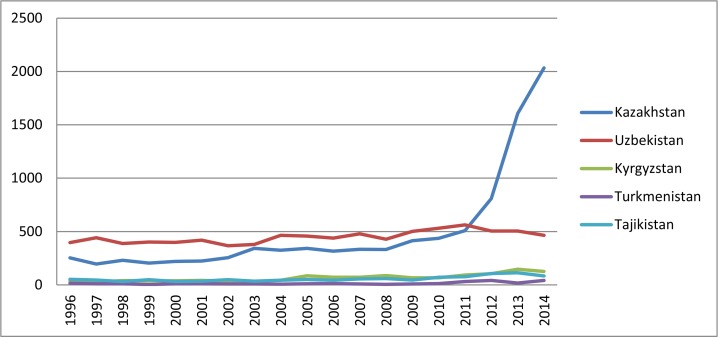
**General scientific publications trends of Central Asian countries for 1996–2014**

While publication productivity patterns are similar across the Central Asian region in 1990s and 2000s, Kazakhstan demonstrated a major increase in the number of publications starting in 2012 (Figure 1). There was a significant difference among countries in the mean number of publications from 1996 to 2014 (F=180.61, *p*<0.0001). Further analysis showed that the mean number of publications was not significantly different between Kazakhstan and Uzbekistan; however, publication rates were significantly different when Kazakhtan and Uzbekistan were compared as a group to Kyrgyzstan, Tajikistan, and Turkmenistan, which had the lowest mean number of publications of all the Central Asian countries at alpha level of 0.05. The breakdown by year shows an increase in the overall number of publications starting in 2012, where Kazakhstani authors published 818 scientific papers in 2012, 1,690 in 2013, and 2,032 in 2014. Kazakhstan’s contribution to the world’s scientific literature increased from 0.03% in 2012 to 0.08% in 2014.

**Figure 2.**
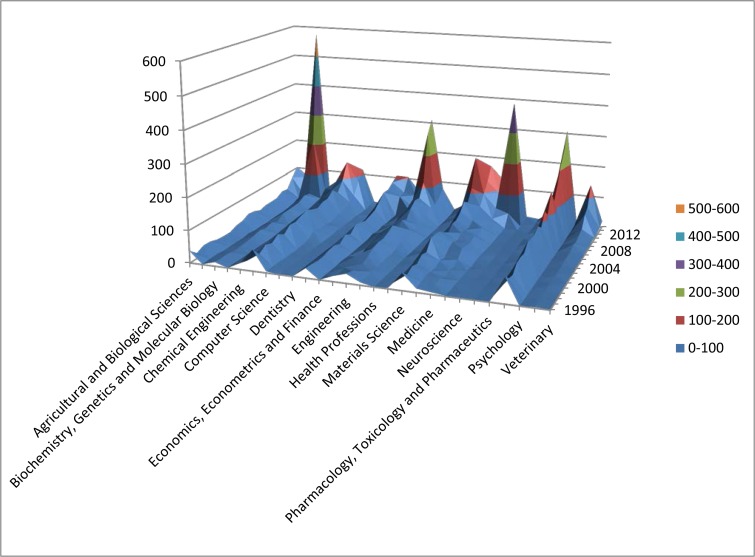
**Change in the number of publications by subject area in Kazakhstan for 1996–2014**

The major contributors to the productivity growth are “Biochemistry, Genetics and Molecular Biology,” “Engineering,” “Multidisciplinary,” and “Physics and Astronomy” (Figure 2). Interestingly, publications in the subject area “Medicine” contributed only 7% of all the publications produced by Kazakhstan during the study period, whereas in the US this percentage was 27%. Overall, according to the SCImago ranking of scientific contribution to health related disciplines, Kazakhstan ranks 126^th^ out of 235 in “Medicine,” 119^th^ in “Public health, environmental and occupational health” out of 223, and 91^st^ in “Epidemiology” out of 216 countries with publications in the respective fields.

From the World Bank data, we can see that Central Asian countries have the lowest expenditures on R&D ranging from 0.12% in Tajikistan to 0.16% in Kazakhstan. In comparison, Russia, Ukraine, and Latvia spent 1.09%, 0.74%, and 0.7%, respectively, on R&D (Table 1). Our analyses showed that GDP and population size are significantly associated with mean number of publications for Central Asian countries (*p*<0.0001). According to World Bank data, Russia, Ukraine, and Latvia have the highest number of individuals involved in the scientific research, as well as the number of published papers per 100,000 population (Table 1) in the post-Soviet region.

## Discussion

Our analysis demonstrated that publication productivity is vastly different among post-Soviet Republics, being relatively low in the Central Asian countries. Among Central Asian countries, the number of publications from Kazakhstan has surged, reaching 2,032 publications in 2014 and 9,632 publications total from 1996 to 2014. However, these numbers are still relatively low compared to other former Soviet Union Republics such as Russia or Ukraine. This poses a significant problem for the global recognition of publication productivity and scientific credibility of scientists in Central Asia, as Scopus referenced publications are oftentimes used as a measure of scientific productivity of scientists in various regions.

A complete analysis of factors associated with differences in publication rates is difficult to produce as publication productivity is driven by complex and multifactorial influences. According to previously published research, the major factors that hinder productivity in developing countries are insufficient funding, low familiarity with foreign languages (i.e. English, in particular), lack of dedicated research centers and international peer-reviewed journals, limited experience with the process of publishing papers in international journals, and insufficient training in the research methods.^10^,^11^

One of the biggest strengths of this study is the ability to comprehensively look at the publication record of the entire Central Asian region over a period of two decades. This approach allows us to objectively evaluate publication trends for multiple disciplines. The limitations of this approach are that it is impossible to evaluate trends prior to 1994, and we are relying on one measure of scientific productivity (publications cross referenced in Scopus).

Another major factor affecting the researchers’ productivity in Kazakhstan and other Central Asian countries is the transition from the Soviet style education and centralized scientific infrustructure system into a more modern and less centralized model of science and education.^12^ Kazakhstan, for example, has become a full member of Bologna Process in 2010.^13^ According to the UNESCO Institute for Statistics,^14^ the number of people with a Bachelor or equivalent degree was 23.1% in Kazakhstan, 0.7% in Kyrgyzstan, 15.2% in Tajikistan, and 15.9% in Turkmenistan. However, since proficiency in foreign languages is not required by higher education institutions of Central Asia, most of the established researchers in post-Soviet Republics struggle to produce high quality English language papers.^5^,^15^

The economic status as well as country population size is important factors that could impact publication productivity and quality. As previously reported, quality of research in developing countries has been found to be dependent on funding available to conduct scientific research.^16^ In our model, GDP and population size were significantly associated with the mean number of publications in the countries under investigation, which suggest that there is need to adjust for these factors when planning research policy.

Another important factor is the number of international peer-reviewed journals published by each country. For example, according to SCImago database in 2012, Russia, Ukraine, and Lithuania have the largest number of Scopus recognized journals with 230, 37, and 38 titles respectively, whereas there are only two Scopus recognized journals, Eurasian Chemico-Technological Journal (eurasianchemtech.vub.ac.be) and Eurasian Mathematical Journal (emj.enu.kz), in the remaining Central Asian countries, and both are published in Kazakhstan. Kazakhstan has recently been pushing to increase its publication rate with the addition of its own scientific journal, the Central Asian Journal of Global Health (cajgh.pitt.edu), which was introduced in 2012.^17^ Starting new peer reviewed English language journals could be the best route for encouraging other countries to develop and advance their own publishing capacity. While this study was not able to capture all factors that may potentially play a role in publication productivity of the regions we explored, including region specific insentives for publication, availability of grnat support, etc, this study was one fo the first attempts to analyze a complex and underinvestigated problem.

After continued lack of focus on research and development which plagued the former Soviet Union Republics in the 1990s, Central Asian countries are rebuilding their scientific capacity.^13^ To our knowledge, Kazakhstan is emerging as a leader in publication productivity in Central Asia as the result of establishing research and development as one of the major goals of national policy. The change in the degree awarding criteria for graduate degrees, effective from early 2011, is an example of such reforms.^13^ According to the new regulations, a candidate for a PhD degree in Kazakhstan must publish at least one document in a non-zero impact factor journal included in ISI Web of Knowledge (Thomson Reuters) or in a journal cited in the Scopus database.^18^ We believe that this is the main reason for the dramatic increase in the number of publications from Kazakhstani researchers which occurred in 2012. As a result, we assume that there is an opportunity to increase the publication rate by introducing new and effective regulations and policies, which can reinforce the necessity of publishing scientific developments in international journals. In addition to publication productivity, our future studies need to explore scientific information dissemination in the region, as these two concepts may be closely related.

Despite the low number of publications in several areas including Public Health and Medicine, the number of articles published in the international journals by Kazakhstani authors has significantly increased, whereas other Central Asian countries demonstrated only a slight increase in publication rates. Consequently, we can assume that policies introduced to increase the publication rate of Kazakhstani researchers had a positive effect. However, we need to consider that the number of publications is still low compared to other former Soviet Union Republics, and, as a result, more efficient and effective actions are needed to increase the number of publications in Kazakhstan and Central Asia in general. We would like to suggest that with improved policies and increased incentives for publishing in English language peer-reviewed journals for Central Asian scientists, we can expect an increase in the number of publications coming from the region.

## Figures and Tables

**Table 1.** Absolute and population adjusted publication rates for 15 former Soviet Union countries for 1996–2014

Country	Number of published documents per country*	Population, total**	GDP per capita, PPP (constant international $)***	Research and development expenditure (% of GDP)***	Researchers in R&D (per million people)***	Number of published documents per 100,000 population*
Armenia	11,741	3,006,154	6,803	0.27	n/a	391
Azerbaijan	9,048	9,537,823	15,754	0.21	n/a	95
Belarus	28,941	9,470,000	16,603	0.70	n/a	306
Estonia	25,458	1,313,645	23,576	2.37	3,485	1938
Georgia	9,821	4,504,100	6,322	n/a	n/a	218
Kazakhstan	9,652	17,289,111	20,772	0.16	652	56
Kyrgyzstan	1,318	5,834,200	2,921	0.16	n/a	23
Latvia	14,403	1,990,351	19,405	0.70	1,904	724
Lithuania	32,137	2,929,323	22,530	0.92	2,756	1097
Moldova	5,506	3,556,400	4,179	0.40	781	155
Russia	701,029	143,819,569	22,570	1.09	3,120	487
Tajikistan	1,118	8,295,840	2,229	0.12	n/a	13
Turkmenistan	284	5,307,188	11,361	n/a	n/a	5
Ukraine	133,650	45,362,900	8,282	0.74	1,253	295
Uzbekistan	8,719	30,742,500	4,412	n/a	534	28

* SCImago Journal and Country rank, 2014

** The World Bank, 2014

*** The World Bank, 2011
